# Microbial degradation of a widely used model polyethylene is restricted to medium- and long-chain alkanes and their oxidized derivatives

**DOI:** 10.1093/ismejo/wraf276

**Published:** 2025-12-16

**Authors:** Ronja Marlonsdotter Sandholm, Gordon Jacob Boehlich, Ørjan Dahl, Ravindra R Chowreddy, Anton Stepnov, Gustav Vaaje-Kolstad, Sabina Leanti La Rosa

**Affiliations:** Faculty of Chemistry, Biotechnology and Food Science, Norwegian University of Life Sciences, 1432 Ås, Norway; Faculty of Chemistry, Biotechnology and Food Science, Norwegian University of Life Sciences, 1432 Ås, Norway; Faculty of Chemistry, Biotechnology and Food Science, Norwegian University of Life Sciences, 1432 Ås, Norway; Norner Research AS, 3920 Porsgrunn, 1432 Ås, Norway; Faculty of Chemistry, Biotechnology and Food Science, Norwegian University of Life Sciences, 1432 Ås, Norway; Faculty of Chemistry, Biotechnology and Food Science, Norwegian University of Life Sciences, 1432 Ås, Norway; Faculty of Chemistry, Biotechnology and Food Science, Norwegian University of Life Sciences, 1432 Ås, Norway

**Keywords:** polyethylene, low-molecular-weight polyethylene, soil microbial communities, alkanes, ketones

## Abstract

Plastics are widely used materials, yet their chemical stability hinders biodegradation, exacerbating pollution on a global scale. Contaminated soils may foster microbes adapted to degrade plastics or derivatives, and these organisms and their enzymes offer promising avenues for the development of biotechnological recycling strategies. Here, two microbial communities originating from soil collected at a plastic-contaminated site in Norway were enriched to select for bacteria involved in the decomposition of a widely used, model polyethylene (low molecular weight, LMWPE; average carbon chain length of 279). We leveraged genome-resolved metatranscriptomics to identify active populations affiliated with *Acinetobacter guillouiae* and *Pseudomonas* sp., showing a suite of upregulated genes (including those encoding alkane 1-monooxygenases, Baeyer–Villiger monooxygenases, and cytochrome P450 monooxygenases) with functions compatible with degradation of medium- and long-chain hydrocarbons and their oxidized derivatives. Spectroscopic, spectrometric and chromatographic analyses revealed the unexpected presence of medium- (C10–16) and long-chain (C17–34) alkanes and 2-ketones in the LMWPE substrate, preventing the erroneous conclusion that the microbial community was degrading the polymeric component. Consistently, only alkanes and 2-ketones of C10–27 were selectively degraded by an *A. guillouiae* isolate, as confirmed by proteomics analyses and substrate characterization following bacterial growth. Besides extending the knowledge on the enzymatic toolbox of soil-associated microbial systems for degrading alkanes and ketones likely arising from abiotic oxidation of polymeric LMWPE, our results provide an advanced compositional characterization of a widely used model “PE” while offering valuable insight to support future studies aimed at unequivocally identifying organisms and their enzymes implicated in PE transformation.

## Introduction

Plastics are synthetic materials composed primarily of long-chain polymers derived from petrochemicals. They have become integral to everyday life due to their light weight, durability, moldability, and cost-effective manufacturing [1]. As a consequence, global production has surged from 464 million tons per year in 2020 to a projected 804 million tons per year by 2050 [2]. Polyethylene (PE) is the most extensively manufactured plastic, constituting ~36% of global plastic production [3]. It is a nonpolar, partially crystalline polymer characterized by its high chemical resistance, low density, and excellent electrical insulation. PE is commonly classified as low-density polyethylene (LDPE), known for its flexibility and transparency, and high-density polyethylene (HDPE), which is more crystalline and rigid [4].

PE-based plastics produced worldwide are recycled, incinerated, sent to landfills, or frequently discarded into the environment. These methods are largely inefficient and energy intensive, with landfilling in particular leading to accumulation of plastic materials with an impact on the surrounding environment [5, 6]. In addition, the mechanical recycling of PE decreases the quality of the material due to thermo-oxidation, crosslinking, and chain functionalization [7]. In the absence of efficient mechano-chemical processes that facilitate reuse of PE, there is an urgent need to explore biological approaches capable of efficiently degrading or transforming plastic waste, thereby enhancing recycling and mitigating its environmental accumulation and impact. PE-based plastics dispersed in plastic dump sites and polluted soils, particularly on the surface, are subjected to prolonged exposure to molecular oxygen, water, sunlight, and UV radiation. These factors can trigger chemical reactions leading to the introduction of polar functional groups (e.g. carbonyl and hydroxyl) onto the polymer backbone, thus rendering PE more accessible to depolymerization, providing a unique setting for microbial adaptation [8]. Therefore, samples from landfills and soil originating from plastic-polluted areas may serve as rich reservoirs of bacteria that have evolved the ability to exploit this novel nutrient niche [9].

To address the plastic pollution challenge, plastic-degrading microorganisms and their enzymes offer promising avenues for the development of biotechnological recycling strategies. In recent years, a multitude of studies have indicated that bacteria belonging to the genera *Acinetobacter*, *Pseudomonas*, *Rhodococcus*, *Brevibacillus*, *Aneurinibacillus*, and *Bacillus*, among others [10–17], possess capabilities to depolymerize PE through different enzyme systems. These potential enzymes belong to various oxidoreductase families, such as alkane hydroxylases, laccases, manganese peroxidases, and monooxygenases [9]. Despite these claims, the mechanism by which PE can be degraded by microbes in natural environments remains controversial [18]. Indeed, interpretation of data on the depolymerization and metabolization of PE is often complicated by the lack of appropriate controls, use of unspecific analytical techniques (e.g. microscopy or infrared spectroscopy), and by the reliance on noncharacterized model plastics, which may contain metabolizable additives and oligomers [19].

In this study, we used a multi-omics approach to functionally characterize two microbial communities enriched on a low-molecular-weight PE (LMWPE), a commonly used model substrate in PE studies. The microbial communities were sourced from a plastic-contaminated site in Norway, and therefore, they faced a prolonged exposure to plastic, including PE, prior to the experiment. We report the isolation of a bacterium, *A. guillouiae* FS11 able to grow on this substrate, and employed proteomics to identify enzymes involved in the depolymerization of components derived from the LMWPE. A combined analytical approach showed that the LMWPE was substantially oxidized “out of the box,” a property not noted by the manufacturer, with consequences on its microbial accessibility. Besides extending the knowledge on the enzymatic basis for degradation of PE-derivatives in soil-associated microbial systems, our findings also offer a cautionary perspective on the increasing number of studies claiming enzymatic degradation of materials with insufficient compositional characterization.

## Material and methods

### Sample collection and microbial profiling using 16S rRNA gene amplicon sequencing

Soil was collected on 1 November 2022 from a plastic dump site in a wooded area in Finnskogen (Flisa, Norway) under a permit from the regional authority of Flisa Municipality. At sampling, large amounts of macroplastic items were present on the surface and buried, mixed in with the soil, having accumulated at this site since the 1970s–1980s without removal. Two locations were sampled: S1 (~5 cm topsoil; 60°40′32.32″N, 12°6′18.98″E) and S2 (~15 cm subsoil; 60°40′32.71″N, 12°6′19.07″E). The original S1 and S2 samples were sent to DNASense ApS (Aalborg, Denmark) for 16S rRNA gene sequencing. Detailed information for DNA extraction, library preparation, sequencing, and data analysis is provided in the Supplementary Material.

### Substrates and growth conditions

PE powder from Sigma-Aldrich (cat. number 427772; weight-average molecular weight (*M*_w_) ~4000 g/mol, number-average molecular weight (*M*_n_) ~1700 g/mol, batch number MKCP961), was used as a model commercial substrate. Because its *M*_w_ is far below that of conventional polyethylene (>100 000 g/mol), we designate the material as an LMWPE to distinguish it from high-*M*_w_, “common” polyethylene throughout the study. Triacontane (C30) and tetracontane (C40) were obtained from Sigma-Aldrich (cat. number 263842 and 87087). D-glucose and sodium succinate were purchased from VWR (cat. number 0188-2.5KG) and Sigma-Aldrich (cat. number S2378), respectively. LDPE granules were obtained from Borealis (grade FT5230) and milled to powder with 200–300 μm particle diameter. Unless stated otherwise, cultures were grown using M9 minimal salts medium (MM) supplemented with a single carbon source [20]. Microbial growth was determined spectrophotochemically by measuring optical density at 600 nm (OD600).

### Selective enrichment of soil samples on LMWPE and alkanes

To minimize carryover of organics from soil, the enrichments were passaged three times in MM containing a single carbon source (LMWPE, C30, or C40). The sequential enrichment protocol was initiated by inoculating MM containing 10 mg/ml LMWPE as a sole carbon source with soil from S1 and S2. These primary cultures were incubated for 10 days; then, 100 μl were transferred to 20 ml of fresh MM with LMWPE to obtain secondary cultures. Tertiary cultures were prepared by inoculating 100 μl of a secondary culture into three separate conditions: 20 ml of fresh MM containing LMWPE (resulting in the cultures indicated as S1LMWPE and S2LMWPE), 20 ml of MM supplemented with 10 mg/ml of C30 (S1C30 and S2C30), and 20 ml of MM supplemented with 10 mg/ml of C40 (S1C40 and S2C40). Incubation conditions in all enrichment phases were 30°C with 200 rpm agitation. At each enrichment step, two controls (i.e. MM without substrate or MM without any microbial source) were also set up and subjected to the same conditions.

### Shotgun sequencing and assembly of metagenomes

Samples from the tertiary communities S1LMWPE, S2LMWPE, S1C30, S2C30, S1C40, and S2C40 were collected in the early stationary phase (Fig. S2) by pelleting at 15 000 g for 10 min. Pellets were resuspended in 800 μl Solution CD1 from the DNeasy PowerSoil Pro kit (QIAGEN, ID: 47014), and DNA was extracted according to the manufacturer’s instructions. DNA size was selected using a Short Read Eliminator XS kit (PacBio, PN: 102-208-200). DNA was quantified using the Qubit dsDNA Broad Range Assay (Invitrogen) on a Qubit 1.0 fluorometer. DNA size was assessed using gel electrophoresis, and purity was evaluated using a NanoDrop One Microvolume UV-Vis spectrophotometer (ThermoFisher Scientific). Sequencing libraries were prepared using the Native Barcoding kit SQK-NBD114.24 (Oxford Nanopore Technologies, UK) following the manufacturer’s instructions. S1LMWPE and S2LMWPE libraries were pooled, as were S1C30 with S2C30 and S1C40 with S2C40. Each pooled sample was then sequenced on a separate R10.4 flow cell on a MinION device (Oxford Nanopore Technologies, UK). POD5 files were base-called and demultiplexed with Dorado v0.5.0 [21], using the super-accurate model (config: dna_r10.4.1_e8.2_400bps_sup@v4.3.0).

The snakemake wrapper Aviary v0.9.1 was used to recover Metagenome-Assembled Genomes (MAGs) [22]. Low-quality MAGs, defined according to the Minimum Information about a Metagenome-Assembled Genome (MiMAG) standards [23], were removed from the dataset. MAGs were taxonomically classified using the Genome Taxonomy Database Toolkit (GTDB-Tk) [24] with the Genome Taxonomy Database (GTDB) database release 220. Functional annotation of the bacterial genomes was performed with DRAM v1.4.6 [25] with the following databases: Uniref90, PFAM-A, KOfam, and dbCAN-V10 (all downloaded in January 2024). Figures were created using R v4.3.3 [26] and ggplot2 v3.5.1 [27].

### Metatranscriptomic analysis

Samples from the tertiary communities S1LMWPE, S2LMWPE, S1C30, S2C30, S1C40, and S2C40 were collected by pelleting at 15 000 g at 4°C in mid-exponential phase after growth on 10 mg/ml LMWPE, 10 mg/ml C30, 10 mg/ml C40 or 20 mg/ml D-glucose. Pelleted samples were shipped to Novogene (Cambridge, UK) for extraction and sequencing of metaRNA. Detailed information for RNA extraction and sequencing is provided in the Supplementary Material. Raw metatranscriptomic reads were quality-checked using FastQC v0.12.1 [28] and filtered using fastp v0.23.4 (−q 20) [29], and ribosomal RNA was removed from the dataset using SortMeRNA v4.3.6 [30]. The filtered metatranscriptomic dataset was pseudo-aligned against the recovered MAGs using Kallisto v0.48.0 [31]. Transcripts per million (TPM) values were log_2_-transformed. Genes with transcripts in two out of three replicates were kept. The remaining missing values were imputed from a normal distribution (width of 0.3 and downshifted 1.8 SD from the original distribution). To identify differentially expressed genes, paired *t*-tests (*P* ≤ .05) were performed using Rstatix v0.7.2 [32], comparing TPM values from C30, C40, or LMWPE to a glucose control. *P*-values were adjusted for multiple testing using the Benjamini–Hochberg False Discovery Rate (FDR) method. Figures were generated using R v4.3.3 [26] packages ggplot2 v3.5.1, cowplot v1.1.3 [33], and ggrepel v0.9.5 [34].

### Isolation of *Acinetobacter guillouiae* FS11 and proteomic analysis

*Acinetobacter guillouiae* was isolated from the S1LMWPE tertiary community following selection on MM containing 10 mg/ml LMWPE and Lysogeny Broth (LB, Sigma-Aldrich, L3522) plates supplemented with 50 μg/ml kanamycin sulfate (Gibco, 11815032). Detailed information for the identification of *A. guillouiae* FS11 is provided in the Supplementary Material. To obtain samples for proteomic analysis, *A. guillouiae* FS11 was cultured in triplicates in MM supplemented with 10 mg/ml LMWPE, 10 mg/ml C30, or 2% v/v sodium succinate. Three sample types were collected in the mid-exponential phase (Fig. S4): planktonic cells, biofilm attached to LMWPE, and secreted proteins. Detailed information for protein extraction, trypsin digestion, and mass spectrometry analysis is provided in the Supplementary Material.

Analysis of mass spectrometry data was done using FragPipe v21.1 (https://fragpipe.nesvilab.org/), with MSFragger v4.0 [35], IonQuant v1.10.12 [36], and Philosopher v5.1.0 [37]. A sample-specific database consisting of the *A. guillouiae* FS11 proteome (4615 proteins) was used. The final database was supplemented with common contaminants (e.g. human keratin, bovine serum albumin, and casein) and contained reverse entries of all library sequences. Carbamidomethylation was used as a fixed modification. Oxidation of methionine and protein N-terminal acetylation were added as variable modifications. Trypsin was used as a digestive enzyme, with a maximum of one missed cleavage allowed. For Label-Free-Quantification (LFQ), IonQuant was applied with FDR-controlled match-between-runs (MBR) enabled. The LFQ intensity values were log-transformed, and a protein was considered “present” if it was detected in at least two of the three biological replicates in at least one condition. Missing values were imputed from a normal distribution. Differential abundance analysis was performed using a paired *t*-test with an FDR-adjusted *P*-value (*P* ≤ .05) in Rstatix v0.7.2 [32] in R v4.3.3 [26]. Figures were generated using the packages ggplot2 v3.5.1 [27], cowplot v1.1.3 [33], and R v4.3.3 [26].

### Protein structure prediction

3D structural models of the top 50 most abundant proteins in the *A. guillouiae* FS11 LMWPE-proteome were generated using AlphaFold3 [38]. Structure similarity searches were performed using FoldSeek [39]. Molecular images, with computation of surface potential, were generated using UCSF ChimeraX v1.10 [40].

### Microscopy

Experimental samples consisted of 10 mg/ml LMWPE derived from MM inoculated with S1LMWPE or S2LMWPE soil communities, or *A. guillouiae* FS11. Control samples consisted of 10 mg/ml LMWPE derived from MM without any microbial source. Scanning electron microscopy (SEM) analysis was conducted using a Hitachi SU5000 microscope (Hitachi High-Technologies Corporation, Japan). The specimens for SEM analysis were prepared by spreading powdered LMWPE samples on an EM-Tec CT12 conductive double-sided adhesive carbon tab (Delta Microscopies, France) and by sputter coating with Platinum with Cressington 208 HR sputter coater (Cressington Scientific Instruments, England). For sputter coating, 20 mA sputter current for 30 s and a sample holder with tilt and rotation was utilized. The platinum-coated specimens were investigated for SEM under high vacuum mode with an SE detector at an accelerated voltage between 1.5 and 3 kV and working distance ~10 mm.

### Substrate characterization

Five analytical techniques were employed to investigate structural and compositional changes in LMWPE following growth of either microbial communities or *A. guillouiae* FS11.

### Fourier-transform infrared spectroscopy

Firstly, we employed Fourier-transform infrared spectroscopy (FT-IR), a widely used method for detecting chemical changes on the surface of the materials analyzed. We sought to identify the appearance of carbonyl groups, absent in the pristine LDPE, and indicated by a peak near 1750 cm^−1^, which signals that the polymer has undergone oxidation. The FT-IR spectrum of the LMWPE and pure LDPE (FT5230, Borealis, Austria) was acquired with a Spectrum Two FT-IR spectrometer (PerkinElmer, Waltham, MA, USA) equipped with a Quest ATR (attenuated total reflectance) sampling module (Specac Ltd, Orpington, United Kingdom). The signals were obtained with 4 cm^−1^ spectral resolution (eight consecutive readings per measurement) in the 4000–550 cm^−1^ range using Spectrum IR software (PerkinElmer, Waltham, MA, USA). The carbonyl index was determined by calculating the ratio of the absorbance peak at 1715 cm^−1^ to the reference peak at 1505 cm^−1^.

### Pyrolysis–gas chromatography–mass spectrometry

Pyrolysis–gas chromatography–mass spectrometry (Py-GC/MS) was employed as a technique capable of characterizing an insoluble substrate without pretreatment, providing insight into the polymer composition and the presence of degradation products. Samples consisting of 150–250 μg of LMWPE or a commercial LDPE (grade 22H594; INEOS Olefins Switzerland) were placed in a deactivated stainless-steel sample cup and introduced into a multi-shot EGA/PY-3030D pyrolyzer (Frontier Laboratories, Japan) coupled to a 7890 N gas chromatograph and a 5975 mass selective detector (Agilent Technologies, USA). The furnace temperature was maintained at 550°C, and the interface between the furnace and the gas chromatography–mass spectrometry (GC-MS) system was set to 300°C. The GC injector was operated in split mode (100:1 ratio) at 300°C. The analysis of volatile products was performed using an Ultra-Alloy metal capillary column containing 5% diphenyl- and 95% dimethylpolysiloxane stationary phase (30 m, 0.25 mm ID, 0.25 μm; Frontier Laboratories Ltd., Japan) using helium as carrier gas at a 1 ml/min flow rate. The GC oven temperature increased from 70°C to 350°C at 20°C/min and was maintained at 350°C for 16 min. The electron ionization (70 eV) mass selective detector was operated with an ion source temperature of 230°C, an interface temperature of 300°C, and a scan range of 29–350 m/z.

Compound identifications were done by matching chromatograms using an F-Search in the Wiley 11th edition/NIST 2017 spectral library. To refine identification, spectral match scores were combined with retention times to differentiate between compounds with similar spectra.

### ^1^H and ^13^C nuclear magnetic resonance spectroscopy

To further characterize the oxidation products detected by FT-IR, nuclear magnetic resonance (NMR) spectroscopy was employed to determine the precise structure of the carbonyl groups. ^1^H-NMR and ^13^C-NMR spectra of LMWPE and PE wax samples were recorded at 328 K using a Bruker AVANCE III HD 400 MHz instrument equipped with a Broadband Observe (BBO) room temperature probe. For sample preparation, 5 mg of pristine LMWPE and a PE wax (Licocene 5301; Clariant AG, Switzerland) were suspended in 0.5 ml CDCl_3_ and transferred to a 5 mm Norell Sample Vault Series NMR tube. Heating these samples to 328 K gave clear solutions. Chemical shifts are reported in ppm with the solvent signals (7.26 ppm for ^1^H/77.16 ppm for ^13^C) as internal standard. The following pulse programs where used: “zg30” for ^1^H, “deptqgpsp.2” for DEPTQ-^13^C, “hsqcetgpsi2” for HSQC, and “hmbcetgpl3nd” for Heteronuclear Multiple Bond Correlation (HMBC).

### Size exclusion chromatography and gas chromatography–mass spectrometry

Size exclusion chromatography (SEC) was used to determine shifts in molar mass distribution, while gas chromatography–mass spectrometry (GC-MS) was employed to detect and determine changes in the amounts of low-molecular-weight compounds in the samples. Three types of samples (untreated LMWPE, LMWPE incubated in MM without any microbial source, and LMWPE incubated in MM with *A. guillouiae* FS11) were prepared to assess the impact of *A. guillouiae* FS11 on substrate composition for subsequent GC-MS and SEC analysis (see Supplementary Methods). The weight-average molecular mass (*M*_w_), number-average molar mass (*M*_n_), and the molecular weight distribution in the LMWPE samples were determined using a gel permeation chromatography (GPC) GPC-IR5 system (Polymer Char, Spain) equipped with four PLgel 20 μm MIXED-A columns (Agilent Technologies, USA). Approximately 4 mg of a pure LDPE (FT5230, Borealis, Austria), LMWPE, and a PE wax (Licocene 5301; Clariant AG, Muttenz, Switzerland) were dissolved in 8 ml of 1,2,4-trichlorobenzene by mixing at 160°C for 3 h. For SEC analysis, 200 μl of the samples were injected into the SEC system. The analysis was performed at 150°C using a high-sensitivity infrared detector and 1,2,4-trichlorobenzene as a mobile phase (1.0 ml/min flow rate). Polystyrene standards with a narrow *M*_w_ distribution and *M*_peak_ (most abundant *M*_w_ in the sample) in the range of 1140–7 500 000 g/mol (Agilent Technologies, USA) were used for calibration.

For GC-MS, extraction of low-molecular-weight compounds was conducted from ~100 mg of LMWPE by mixing with 3 ml of ethyl acetate in PTFE/Silicone septum sealed glass vials. The vials were heated at 95°C for 1.5 h and the liquid fraction filtered through a 0.2 μm Teflon syringe filter and subjected to GC-MS analysis. GC-MS analysis was carried out using an Agilent 6890 N gas chromatograph (Agilent Technologies, Inc., USA) coupled to an Agilent 5973 Network Mass Selective Detector (Agilent Technologies, USA) and a Gerstel MPS2 Autosampler (GERSTEL GmbH & Co. KG, Germany). The gas chromatograph was equipped with 30 m Zebron ZB-5MSPlus column having a 250 μm internal diameter and 0.25 μm coating thickness (Phenomenex, USA). The temperature of the column increased from 60°C to 300°C at a heating rate of 10°C per minute with a total run time of 45 min. Helium with a purity grade of 6.0 was used as a carrier gas at a flow rate of 3 ml/min. The mass spectrometer was operated in electron ionization mode at 70 eV. Mass spectra and the total ion chromatograms were obtained by automatic scanning a mass range (*m*/*z*) of 33–720. Three runs per sample (*n =* 3) were performed. The volatile components were identified by comparing the mass spectrum with those available in the Wiley 11th edition/NIST 2017 spectra library. Compounds were quantified by calculated response factors. Response factors were calculated by using the reference compounds BHT (butylated hydroxytoluene) and Tinuvin 120 (2′,4′-Di-tert-butylphenyl 3,5-di-tert-butyl-4-hydroxybenzoate), as they give differences in the responses between lower- and higher-molecular-weight components. The analyzed LMWPE samples were confirmed to contain no compounds chemically similar to Tinuvin 120 or BHT.

## Results

### Microbial communities from a plastic-enriched site include metabolically active bacteria that grow on LMWPE but not on LDPE

As part of an effort to discover and characterize microbes and their enzymes able to degrade LDPE and LMWPE, soil samples were obtained from a site that has been exposed to plastic waste since the 1970s. Samples were collected from two different locations and depths and subjected to 16S ribosomal RNA (rRNA) gene amplicon sequencing, which revealed species previously associated with the degradation of various plastics and alkanes (Supplementary Results and Fig. S1).

An enrichment protocol by sequential passaging was employed in order to select microbial species specifically involved in the degradation of LDPE, LMWPE, and two distinct alkanes (triacontane and tetracontane). The two alkanes were used as substrates, given that they may trigger catabolic systems also used for PE degradation. Cultures showed growth on LMWPE, triacontane, and tetracontane (Fig. S2). In contrast, no growth was observed when LDPE was provided as the sole carbon source.

**Figure 1:**
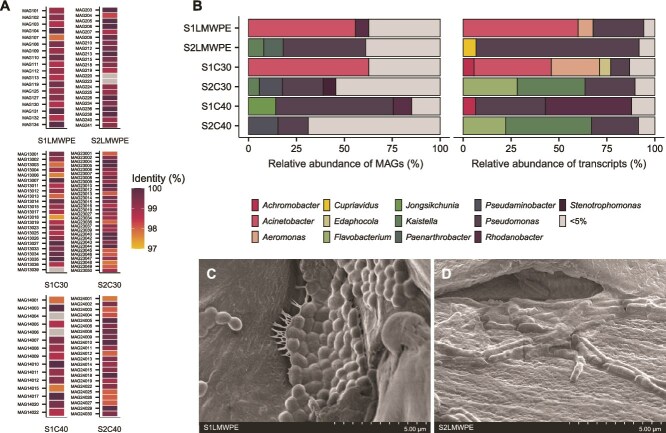
Relative abundance and activity of MAGs in the LMWPE, triacontane (C30), and tetracontane (C40) enrichment cultures as well as their detection in the original soil communities. (A) A list of all high- and medium-quality MAGs reconstructed from the different enrichment cultures and their identification in the original S1 and S2 soil communities. The detection is based on the alignment of the 16S rRNA gene sequence of each individual MAG to the OTUs detected in S1 and S2 (Fig. S1). The 16S rRNA gene detection is colored based on the % identity of the gene alignment. At a 97% identity level to OTUs, 96% of the MAGs were detected in the S1 and S2 soil samples. (B) Relative abundance of MAGs (grouped at genus level and based on the assembled genome fraction) in the LMWPE, triacontane (C30), and tetracontane (C40) enrichment cultures and the relative abundance of total transcripts per million (TPM) for each MAG within each community. Genera with a relative abundance of <5% are grouped together. (C) and (D) show SEM images of the LMWPE surface following growth with the S1LMWPE and S2LMWPE communities, respectively. Each community exhibits a distinct morphology of bacteria attached to the LMWPE surface.

Long-read metagenomic sequencing of the enrichment cultures yielded a total of 84 high-quality and 46 medium-quality dereplicated MAGs (Fig. S3). The use of long-read DNA sequencing ensured that 127 MAGs encoded full-length 16S rRNA genes, which enabled searches for the occurrence of these bacteria in the amplicon dataset generated from the original soil samples. At a 97% identity cut-off when comparing the 16S rRNA gene sequences derived from the MAGs in the enrichment cultures to Operational Taxonomic Units (OTUs), 125 of 130 MAGs were detected in the original S1 and S2 soil microbial communities (Fig. 1A). Of the undetected MAGs, three of five MAGs were missing a 16S rRNA gene. The S1LMWPE and S1C30 enrichments were characterized by the dominance of MAG107 (55.8%) and MAG13003 (62.8%), respectively, both taxonomically identified as *A. guillouiae* (Fig. 1B, Table S1A). *A. guillouiae* was absent from the S1C40 community, suggesting its limited capacity to metabolize alkanes with a chain length > 30. The S2LMWPE and S1C40 consortia were characterized by a high relative abundance (42%–62%) of MAGs affiliated to the genus *Pseudomonas*. Scanning electron microscopy (SEM) provided an initial qualitative insight into the distribution and morphological diversity of microbial cells with the ability to adhere to the substrate. Species with distinct morphologies were observed in the S1LMWPE and S2LMWPE communities (Fig. 1C and D). In both samples, microorganisms formed dense and slimy clusters on the LMWPE particles, resembling biofilms. Additional 16S RNA gene sequencing of the bacteria attached to the LMWPE surface confirmed the presence of *Acinetobacter* and *Pseudomonas*, respectively, in agreement with their prevalence in the corresponding enrichment community.

A genome-resolved metatranscriptomics approach allowed us to identify metabolically active MAGs and their upregulated genes in response to LMWPE, triacontane, or tetracontane to a control where glucose was used as the sole carbon source. Intriguingly, transcripts abundance (relative to the total TPMs) for each MAG in the communities did not always reflect their abundance in the enrichments (Fig. 1B). In the S1LMWPE community, the most abundant, and most active MAG, was MAG107 (*A. guillouiae*) (Fig. 1B). The second most active MAGs, MAG108 (*Pseudomonas* sp.), and MAG134 (*Aeromonas*), were not prevalent MAGs in the enrichment community (3.2% and 0.8%, respectively). As for the S2LMWPE community, MAG238 (*Pseudomonas* sp002029345) and MAG206 (*Pseudomonas* sp.)were highly active, with MAG238 not being the most predominant *Pseudomonas* in the enrichment (8.3%). This observation applies to all the enrichments, where the most abundant MAGs were not necessarily the most active, highlighting the need for functional data to accurately characterize true metabolism of substrates by microbial community members that may be dormant or slow growers.

**Figure 2:**
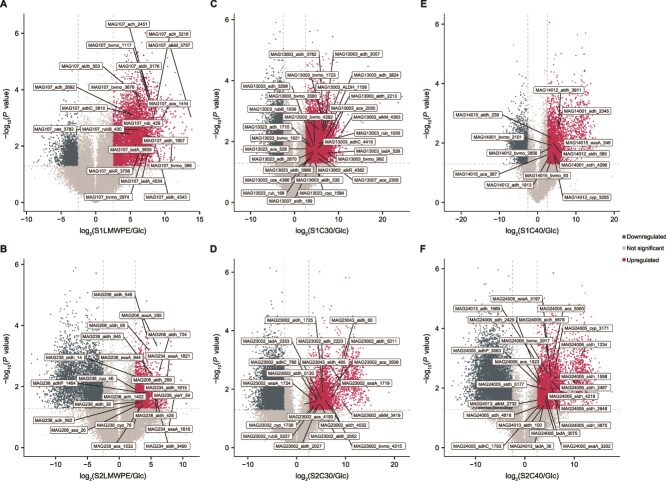
Volcano plot indicating different transcripts in the LMWPE, triacontane (C30) and tetracontane (C40) degrading communities that displayed a large magnitude of fold-changes in TPMs and high statistical significance (−log_10_ of *P*-values, *t*-test) compared to the same communities grown on glucose. (A) and (B) display upregulated genes in the S1LMWPE and S2LMWPE tertiary communities, respectively. (C) and (D) show upregulated genes in the S1C30 and S2C30 tertiary communities, respectively. (E) and (F) show the upregulated genes in the S1C40 and S2C40 tertiary communities, respectively. KEGG Orthology IDs were used to identify and highlight genes known to be involved in alkane degradation, 2-ketone degradation, and β-oxidation of fatty acids. The dashed horizontal line denotes the *P*-value cut-off (*P* < .05), and the dashed vertical lines denote the log_2_-fold change cut-off (<−2.5 and >2.5). Source data are provided in Table S1B–G.

Closer examination of the metatranscriptome showed that, in the S1LMWPE community, MAG107 (*A. guillouiae*) accounted for all upregulated genes involved in the alkane terminal oxidation pathway, including those coding for an alkane 1-monooxygenase (*alkM*) and the electron-transfer components rubredoxin (*rubB*) (Fig. 2A). In addition, several genes annotated as encoding flavin-containing monooxygenases (FMOs) were upregulated. Consistent with the fact that true FMOs are rarely reported in bacteria [41], the structure of these proteins displayed high similarity (data not shown) to flavin-dependent monooxygenases known as Baeyer–Villiger monooxygenases (BVMOs). Accordingly, the *fmo* genes identified in our dataset are referred to as *bvmo*. Although MAG107 appears to be the principal driver of substrate degradation, other MAGs including MAG134 (*Aeromonas aquatica*) and MAG108 (*Pseudomonas* sp.) displayed upregulated genes coding for enzymes involved in β-oxidation of fatty acids (Table S1B). In the S2LMWPE community, several MAGs exhibited upregulated genes encoding enzymes contributing to alkane degradation (Fig. 2B). Here, MAG238 (*Pseudomonas* sp002029345), MAG206 (*Pseudomonas fluorescens*), and MAG234 (*Pseudomonas veronii*) show increased expression of genes coding for aldehyde dehydrogenases (*aldH*) and alcohol dehydrogenases (*yiaY*, *adhP*, *ADH*, and *exaA*). In this community, genes coding for cytochrome P450s (CYPs), which have been associated with both terminal and subterminal hydroxylation of alkanes [42], were upregulated in MAG238, suggesting its potential role as main degrader (Table S1C).

The S1C30 community was dominated by MAG13003 (*A. guillouiae*), which exhibited upregulation across the full suite of genes involved in alkane degradation (Fig. 2C). In addition, MAG13023 (*Achromobacter* sp.) upregulated genes encoding alcohol dehydrogenases and aldehyde dehydrogenases, as well as a CYP450, suggesting a potential role in alkane transformation. Upregulated genes coding for BVMOs were detected in MAG13003 (*Achromobacter* sp.), potentially oxidizing 2-ketones generated by CYP450’s activity. Although MAG13023 was not one of the most active MAGs in the community (Fig. 2C), this MAG and MAG13003 seem to share the role as main degraders, whereas MAG13012 (*Achromobacter kerstersii*), MAG13007 (*Aeromonas aquatica*), and MAG13006 (*P. putida*) have genes in the β-oxidation pathway upregulated (Table S1D). In the S2C30 community, MAG23002 (*Pseudomonas* sp.) showed upregulated genes in the alkane degradation pathway, including *alkM*, the long-chain alkane monooxygenase *ladA*, and *cyp450*, as well as the β-oxidation pathway, suggesting its role as the main degrader (Fig. 2D). MAG23043 (*Kaistella soli*) displayed upregulated genes encoding enzymes involved in the β-oxidation pathway (Table S1E).

In the S1C40 community, MAG14012 (*Achromobacter* sp.) upregulated genes encoding a CYP*,* as well as genes encoding enzymes involved in the full alkane degradation pathway and the β-oxidation pathway were detected (Fig. 2E, Table S1F). Other active MAGs (MAG14015; *Pseudomonas* sp, MAG14001; *A. guillouiae* and MAG14011) lacked the initial alkane hydroxylation step but showed upregulation of genes coding for enzymes involved in the subsequent alkane oxidation and β-oxidation pathway, as well as MAG14001 upregulated a gene coding for a BVMO. In the S2C40 community, MAG24005 (*P. fluorescens*), and MAG24013 (*P. veronii*) showed upregulated genes coding for AlkM and LadA, with MAG24005 also upregulating a gene coding for a CYP450 (Fig. 2F, Table S1G).

Overall, comparison of the community transcriptional response in LMWPE versus S1C40 and S1C30 revealed no upregulated genes in LMWPE that encode enzymes potentially capable of cleaving the C-C bonds of the polymeric component.

### Low-molecular-weight polyethylene (Sigma-Aldrich PE) is oxidized at the chain ends

**Figure 3:**
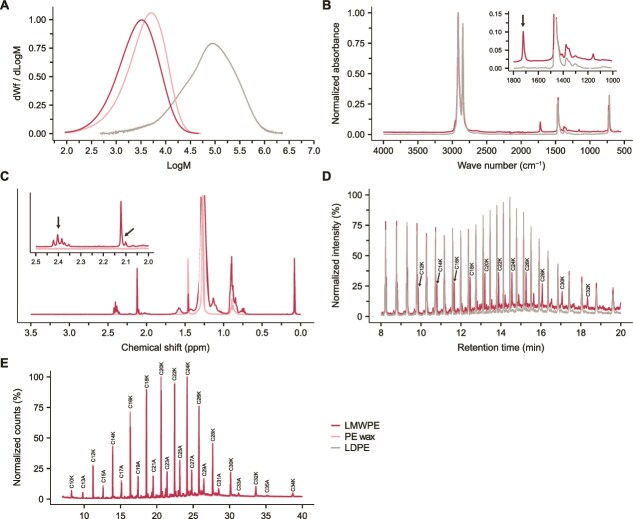
Characterization of LMWPE. (A) SEC analysis of LMWPE, a reference LDPE, and a PE wax. The molar mass distribution of LMWPE is different from that of the PE wax and LDPE. (B) Overlayed FTIR spectra of the LMWPE and a reference LDPE. Compared to LDPE, the strong additional peak at ≈1722 cm^−1^ (black arrow) clearly indicates oxygen functionalities in the LMWPE (i.e. carbonyl groups in ketones or aldehydes). (C) NMR spectra of LMWPE. The singlets at 2.10–2.12 ppm and the triplet between 2.35 and 2.42 ppm (black arrows) confirm the presence of a carbonyl at the second carbon. (D) Overlaid Py-GC–MS chromatograms of LMWPE and LDPE. In the LMWPE, additional peaks were observed that were absent in the LDPE. These peaks correspond to ketones (K) and are labelled according to the chain length as number of carbons (C) (see Table S2D for details about peak annotation). (E) GC-MS chromatograms of LMWPE ethyl acetate extracts, showing alkanes and 2-ketones between 10 and 35 carbons in chain length. Peaks are labeled by the chain length as number of carbons (C) as well as compound class, with “A” denoting alkanes and “K” denoting ketones. Peak annotations are also available in Table S2E. The three shades of pink represent technical triplicates of the LMWPE ethyl acetate extracts. Reference LDPE data for (A) and (B) were obtained from Stepnov *et al.* [55]. Source data can be found in Table S2A–E.

Many studies have reported microbial degradation of the Sigma-Aldrich PE (i.e. catalog number 427772) [15, 43–50], referred to as LMWPE in this study, a phenomenon reproduced by our enrichment cultures (Fig. S2). Whereas no growth on pristine LDPE and no differentially expressed genes that could be linked to enzymes able to interact with the polymeric substrate was observed, it was of interest to perform a deeper analysis of the LMWPE material to identify the constituents that may be responsible for sustaining microbial growth. SEC analysis showed that the LMWPE *M*_w_ is ~3900 g/mol (average carbon chain length of 279), which is similar to what is reported by the manufacturer (~4000 g/mol). This is indeed substantially lower than a reference LDPE, with M_w_ of 97 300 g/mol, and slightly lower than a PE wax (~5400 g/mol) (Table S2A). To determine the putative presence of functional groups from oxidized derivatives, LMWPE and LDPE were subjected to FTIR analysis (Fig. 3B). A strong peak at ≈1722 cm^−1^ was observed in the LMWPE sample corresponding to stretching of a carbonyl group in an aldehyde or a ketone, accompanied by additional much weaker peaks at ≈1409 and ≈1158 cm^−1^. The LDPE sample showed no sign of oxidation. To identify the type and extent of oxidation of the LMWPE, the polymer was analyzed by NMR. To determine the presence of ketones in the substrate, as well as the positions of the carbonyl groups, the LMWPE structure was elucidated using 1D and 2D ^1^H and ^13^C-NMR (Fig. 3C). The ^1^H-NMR spectrum displayed a large singlet at 1.28 ppm, which is characteristic of the CH_2_-groups within the hydrocarbon backbone of PE. As expected, this peak was present in both the PE wax and the LMWPE samples. Additional signals from the LMWPE were detected between 2.00 and 2.50 ppm; the chemical shift of these signals is typical for protons located next to carbonyls. The singlets at 2.10 and 2.12 ppm are characteristic of CH_3_ groups next to a carbonyl. The two triplets at 2.37 and 2.40 ppm indicate CH_2_ groups next to a carbonyl. The ^1^H-^13^C-HMBC-spectrum showed correlations from the triplet at 2.40 ppm and the singlet at 2.12 ppm to a carbonyl signal at 208.7 ppm, confirming that these signals stem from a methyl ketone (2-ketone). The smaller triplet at 2.37 ppm showed a correlation to a carbonyl signal at 211.3 ppm, indicating this triplet stems from in-chain ketones. The small singlet at 2.10 ppm showed a correlation to a carbonyl at 212.5 ppm, which stems from a 2-ketone next to a branching point. At 9.77 ppm, the ^1^H-NMR-spectrum also showed a triplet just above the noise level, indicating that trace amounts of aldehydes are also present. The NMR data also provide a means to quantify the degree of oxidation. Based on quantification by ^1^H-NMR, approximately 6‰ of all individual carbons in the LMWPE are covalently linked to an oxygen atom. Of these, 84% are 2-ketones, suggesting that the majority of the chains in the substrate have oxygen functions. The Py-GC-MS data revealed the presence of analytes in the LMWPE sample that were not observed in the LDPE control sample (Fig. 3D). These peaks were identified as 2-ketones between 12 and 32 carbons, using a reference library (Table S2D). GC-MS analysis confirmed the presence of these small molecules by identifying hydrocarbons, including medium and long-chain alkanes with 13–35 carbons and 2-ketones with 10–34 carbons in the LMWPE samples (Fig. 3E, Table S2E). The presence of short 2-ketones in the LMWPE introduces oxygen functionality, potentially increasing the bioavailability of the plastic compared to pristine LDPE.

### The bacterial isolate *A. guilloiae* FS11 catabolizes low-molecular-mass alkanes and 2-ketones present in LMWPE

**Figure 4:**
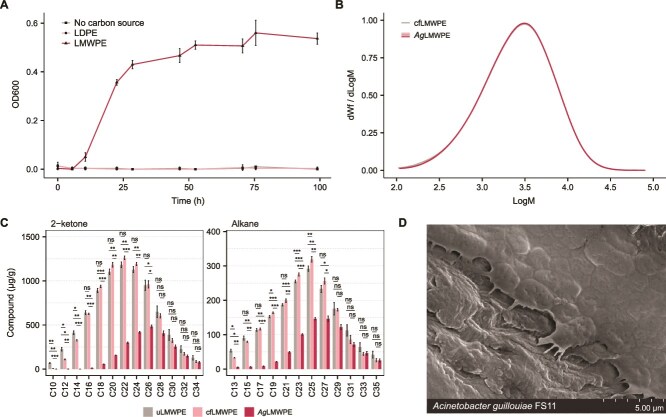
Growth profiles of *A. guillouiae* FS11 on LMWPE and subsequent characterization of the substrate. (A) Growth of *A. guillouiae* FS11 in minimal medium (MM) without any carbon source or supplemented with either LMWPE or LDPE. Data are averages ± standard deviations (error bars) of three biological replicates. (B) SEC chromatograms of LMWPE following incubation with MM (cfLMWPE) or MM with *A. guillouiae* FS11 (*Ag*LMWPE). One control sample (cfLMWPE) and three replicates (*Ag*LMWPE) were set up. Sample-specific chromatograms are shown. Note that chromatograms are superimposing in the high molecular weight regions, indicating no degradation of polymeric fraction of LMWPE substrate, as the molar mass distribution would shift to the left. (C) GC–MS results of LMWPE showing the reduction of each compound in the LMWPE samples after growth of *A. guillouiae* FS11 (*Ag*LMWPE). Compound loss is expressed relative to the amount of the same compound detected in untreated LMWPE (uLMWPE) compared to LMWPE incubated with MM (cfLMWPE) and an untreated control (uLMWPE). Data are averages ± standard deviations (error bars) of three biological replicates, and significance levels are displayed for pairwise *t*-test [not significant (ns), *P* ≤ .05 (^*^), *P* ≤ .01 (^**^), *P* ≤ .001 (^***^) and *P* ≤ .0001 (^****^)]. Non-biological losses of 2-ketone of C10–14 and alkanes of C13 are attributable to vaporization and/or solubilization during incubation. (D) SEM image of *A. guillouiae* FS11 adhering to the surface of LMWPE. Source data for SEC and GC-MS can be found in Table S2F and G.

To gain deeper insight into the bacterial metabolism of LMWPE constituents, we isolated the dominant species from the metagenomic analysis, namely, *A. guillouiae* FS11. Although *A. guillouiae* FS11 did not grow on pristine LDPE (Fig. 4A), its growth on LMWPE was rapid for an insoluble substrate, showing an increase in OD600 from 0.00 to 0.56 after 75-h incubation (Fig. S4). The molecular mass distribution curve obtained in the SEC analysis for LMWPE after bacterial growth (*Ag*LMWPE) closely overlapped with that of the abiotic control sample (cfLMWPE), providing no evidence for degradation of polymeric PE substrate (Fig. 4B). GC-MS quantification of the low-molecular-mass components showed depletion of the smallest alkanes and 2-ketones and substantial reduction in medium-length compounds (10–27 carbons in length) in *Ag*LMWPE, compared to the control samples (Fig. 4C). The LMWPE particles that were only exposed to MM (cfLMWPE) also showed a partial reduction in C10–14 alkane and C13 2-ketone oligomers (Fig. 4C), likely due to vaporization or solubilization during the treatment period. Conversely, the uLMWPE sample remained untreated, accounting for the higher concentrations (20.66%–88.99%) of these low-molecular-weight alkanes and ketones. Similar to the observations made for enrichment cultures growing on LMWPE (Fig. 1C and D), *A. guillouiae* FS11 formed biofilms on the surface of LMWPE particles (Fig. 4D).

### *A. guillouiae* FS11 may use Baeyer–Villiger monooxygenases for 2-ketone conversion

**Figure 5:**
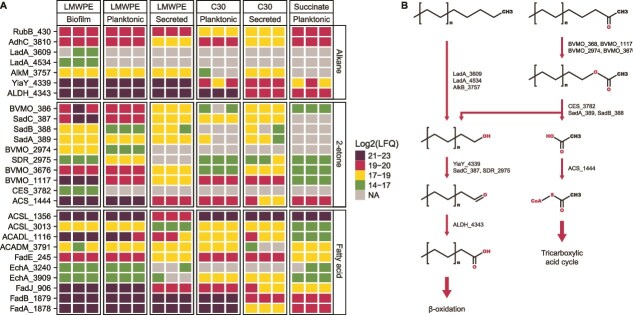
Selected differentially abundant proteins in the *A. guillouiae* FS11 proteome and proposed metabolic pathway for degradation of 2-ketones and alkanes in LMWPE. (A) LFQ intensities of the known and proposed enzymes in the pathways for terminal alkane oxidation, 2-ketone degradation, and β-oxidation of fatty acids. Missing values are shown in gray. (B) Proposed pathway enabling metabolism of alkanes and 2-ketones present in LMWPE, with chain lengths ranging from 10 to 27. Alkanes are terminally oxidized, while 2-ketones are oxidized by BVMOs, generating esters. These esters are further hydrolyzed by an esterase into alcohols and acetate. The alcohol can be oxidized by an alcohol dehydrogenase to an aldehyde, which is subsequently converted by an aldehyde dehydrogenase into a fatty acid that can enter the β-oxidation pathway. Acetate can enter the tricarboxylic acid (TCA) cycle.

To identify the enzymes involved in the degradation of the oligomeric alkanes and 2-ketones in LMWPE, we conducted a proteomic analysis of secreted, planktonic, and biofilm-derived proteins produced by *A. guillouiae* FS11 during growth on LMWPE, triacontane, or sodium succinate. Enzymes involved in alkane degradation, 2-ketone degradation, and β-oxidation were generally more abundant under LMWPE conditions (Fig. 5A). In the biofilm-derived proteome, all known enzymes involved in alkane degradation, as well as the putative enzymes involved in 2-ketone oxidation, were detected (Fig. 5A). Two LadA (LadA_3609, LadA_4534) were exclusively detected in the LMWPE-biofilm proteome. AlkB_3757 was detected in all proteomes, except for the succinate control. Finally, YiaY_433 and ALDH_4343 were among the most abundant proteins detected in the LMWPE-associated (biofilm, planktonic, and secreted) and C30 (planktonic) proteomes (Fig. 5A), indicating that enzymes involved in the complete alkane degradation pathway are active under these conditions.

Several differentially abundant BVMOs that may be actively transforming 2-ketones were identified in the LMWPE and C30 proteomes (Fig. 5A). All these BVMOs exhibit high structural and sequence similarity to characterized BVMOs (Fig. S6, Table S1J). These included the BVMO_386, encoded by a gene associated with a cluster that was confirmed via BLASTn analysis to correspond to the *sad* gene cluster of *Acinetobacter sp.* strain NyZ410 [51]. FoldSeek predictions using AlphaFold3 models indicated that BVMO_386 corresponds to SadD, a BVMO known to convert aliphatic ketones into esters [51]. Additional BVMOs were detected at high abundance either exclusively in the LMWPE proteome (BVMO_2974), or in both the LMWPE or C30 proteome (BVMO_3676, BVMO_1117). The presence of multiple BVMOs in the proteome may reflect the need to accommodate the 2-ketones of different chain lengths present in the LMWPE (Fig. 4C), as such protein redundancy is commonly considered a microbial strategy to expand substrate range [52]. The esterases SadA_389 and SadB_388, together with the carboxylesterase CES_3782, exhibited significantly higher abundance in the proteomes derived from LMWPE and C30 compared to the control conditions (Fig. 5A). The hydrolysis products (alcohol and acetate) derived from the activity of these enzymes follow distinct fates: the alcohol can enter the terminal alkane oxidation pathway as an intermediate and is sequentially converted into a fatty acid that undergoes β-oxidation, whereas the acetate is converted into acetyl-CoA by the acetyl-CoA synthetase ACS_1444 and enters the tricarboxylic acid cycle (TCA) (Fig. 5A).

Collectively, the detection of the enzymes described above (Fig. 5A), in combination with the depletion of both alkanes and 2-ketones (Fig. 4C), indicate that the small molecules derived from the LMWPE can be consumed through terminal alkane oxidation and 2-ketone degradation, which is a shorter version of the established pathway for subterminal oxidation of alkanes (Fig. 5B). This corresponds to the known route of 2-ketone oxidation, where SadD (a BVMO) and SadA/SadB (esterases) convert 2-ketones into primary alcohols and acetate [51].

## Discussion

### Understanding microbial degradation of PE requires thorough characterization of the substrate

Biotechnological conversion of PE to useful products, such as platform chemicals or microbial single-cell proteins, is an important research area given the unfathomable amounts of PE waste available and continuously accumulating on the planet. Indeed, many studies exist where PE is reported to be microbially or enzymatically degraded [53, 54], but few of these have been reproduced by others, and some attempts at reproduction have failed [55]. Recently, several cautionary articles and reviews have been published that urge research to be more careful in experimental design and interpretation of results [18, 55, 56]. One common misunderstanding arises from the use of model PE substrates that do not have the properties of a typical industrial-grade PE. A very good example is the LMWPE used in the present study, which is marketed as “PE” by the supplier (Sigma-Aldrich). This product has been used in a multitude of studies [15, 43–50], many of which report microbial PE-degradation without providing evidence of C-C bond cleavage in the polymer backbone. Thus, proposed enzymes such as laccase-like multicopper oxidases (LCMOs) [50], CYP [50], AlkB [46, 50], or glutathione peroxidase (GPx) [46] likely cannot perform PE backbone cleavage and may instead act on oligomeric PE-derivatives in the substrate.

From a polymer chemist’s standpoint, the Sigma-Aldrich PE is an LMWPE (*M*_w_ ~ 3900 g/mol), and such a polymer cannot be compared to a typical commercial PE, which has *M*_w_ often exceeding 100 000 g/mol, where the high molecular weight is what provides the high chemical and structural robustness. Our data clearly show that bacteria like *A. guillouiae* F11, as well as soil microbial communities, can efficiently utilize LMWPE as a sole carbon source by metabolizing the alkane oligomers and 2-ketones present in the substrate, while not interacting with the polymeric component (Fig. 4 and Fig. S2). In our commercial LDPE sample, no such oligomers were present (Fig. 3); thus, neither the *A. guillouiae* F11 isolate nor the soil-derived microbial communities were able to grow (Fig. S2 and Fig. S4). It is not unlikely that the mechanism of LMWPE utilization in other studies, where the LMWPE is mistaken for a compound that has the properties of high-molecular mass PE (even referred to as LDPE instead of LMWPE [43, 45, 47, 48]), is the same as observed in our study. A second key observation made in the present study is the abundant presence of 2-keto groups in the LMWPE chains (Fig. 3), a feature absent from the supplier’s product specification, that could increase the material’s biodegradability. We are not the first to identify oxidation of the LMWPE. Zampolli *et al.* also performed GC–MS on the material (most likely a different batch) and reported the presence of multiple different oxygen-containing functional groups, including 2-ketones [49]. These observations suggest that it is not only the product purchased for the present study that is oxidized but possibly a larger batch of the product. FTIR analysis of two independently purchased LMWPE samples (batch numbers MKCT5408 and MKCP961) confirmed the same oxidation pattern (results not shown). Carbonyl groups can be introduced into PE, including the low-molecular-weight fractions, through, i.e. chemical [57], thermal [58], and photo-oxidation [59], supporting the idea that these oxidized components are derived from the original LMWPE material.

As demonstrated in our study, rigorous characterization of the material used in biodegradation studies is essential to make claims of true PE depolymerization. Solely relying on indirect methods, such as FT-IR spectroscopy or SEM imaging [60–62], does not demonstrate the full extent of PE metabolization [18, 63].

### Metabolic insight into the consumption of LMWPE derivatives by soil microbial communities and a bacterial isolate

The mechanism by which LMWPE-derived oligomeric alkanes and 2-ketones are consumed can give valuable insight into how PE waste potentially could be enzymatically depolymerized, given that the material is pretreated to reduce molecular weight, e.g. by oxidation. When searching for microorganisms with the capability to depolymerize PE, or oxidized PE components and derivatives, it is reasonable to focus on natural communities that have been exposed to plastic pollution over many years.

In this work, an enrichment experiment was established using an oxidized LMWPE and soil communities collected from a site contaminated with weathered plastic waste. Shotgun metagenomic profiling showed that *Acinetobacter* and *Pseudomonas* spp. became abundant in the enrichment cultures on LMWPE (S1LMWPE and S2LMWPE), or on alkanes such as triacontane (S1C30 and S2C30) and tetracontane (S1C40 and S2C40) (Fig. 1B). Members of the genera *Pseudomonas* [64, 65] and *Acinetobacter* [52, 66] are some of the most studied alkane-degrading microorganisms, and have been proposed to be able to adhere and/or degrade PE [12, 67–69].

Genome-centric metatranscriptomics showed that the microbial communities in the enrichment cultures could actively metabolize LMWPE as the sole carbon source, whereas no growth was observed in the pristine LDPE (Fig. S2). LMWPE contains shorter hydrocarbons, alkanes, and 2-ketones (Fig. 3), which served as substrates for the microbes [70]. Genes coding for enzymes for terminal oxidation of alkane, including the monooxygenases LadA and AlkB, were upregulated in the S1LMWPE, S1C30, S2C30, and S2C40 enrichments (Fig. 2). In the S2LMWPE and S1C40 enrichments, upregulated CYP genes may allow subterminal hydroxylation of alkanes (Fig. 2C and D), enabling the microorganisms to degrade alkanes without AlkB or LadA [42]. Several genes encoding BVMOs were upregulated on LMWPE, C30, and C40 (Fig. 2). The BVMO family comprises a diverse range of xenobiotic-metabolizing enzymes, which use NADPH as a cofactor and FAD as a prosthetic group. This family includes BVMOs that can also play a role in the subterminal oxidation of alkanes [71]. Here, an alkane monooxygenase introduces a hydroxyl group at a subterminal position of the alkane chain, which is further oxidized to a ketone by an alcohol dehydrogenase. The carbonyl group in the ketone can then be converted into an ester group by a BVMO [70]. The predicted BVMOs detected in the enrichment cultures can contribute to the conversion of 2-ketones, derived from the LMWPE, or subterminally hydroxylated alkanes, into esters.

Following isolation of the dominant species involved in LMWPE degradation, *A. guillouiae* FS11, proteomics and substrate analyses further demonstrated that it metabolized the smaller compounds (alkanes and 2-ketones of chain lengths between 10 and 27 carbons) in the LMWPE but is not able to cleave the polymeric backbone (Fig. 4). Proteomic results revealed that *A. guillouiae* FS11 metabolizes LMWPE components via terminal alkane oxidation, β-oxidation, and the TCA cycle. The isolate showed abundant AlkB, LadA, Adh, YiaY, ALDH, and several BVMOs, including BVMO_2974, uniquely detected in LMWPE-derived proteomes (Fig. 5A). BVMOs may actively interact with the 2-ketones and alkanes present in the oxidized LMWPE via the subterminal oxidation pathway (Fig. 5B). Overall, these results are consistent with previous work in which an enzyme cascade comprised of a catalase-peroxidase, an alcohol dehydrogenase (Adh), a BVMO, and a lipase/esterase was applied to depolymerize LMWPE that had been chemically pretreated to introduce hydroxyl or ketone groups [57]. While bacterial BVMOs have been shown to be involved in the conversion of secondary alcohols of C10–12 into primary alcohols [57, 72], the exact role of the three putative BVMOs from *A. guillouiae* FS11 in processing secondary alcohols derived from 2-ketones of varying chain lengths in the LMWPE employed in this study remains to be elucidated through biochemical characterization.

## Conclusion

Our study provides a valuable approach for studying how microbial communities interact with PE and PE-derivatives, and results support two main conclusions. First, advanced substrate characterization before and after microbial growth is essential to convincingly support assumptions of microbes being able to break down the PE backbone and subsequent mineralization. Second, the soil-derived microbial community enrichments and isolated *A. guillouiae* FS11 could efficiently utilize shorter-chain and oxidized-derivatives of PE, whereas no evidence of degradation of longer chain, pristine LDPE was obtained. These results, in combination with the lack of biochemically characterized enzymes which act on polymeric PE [73], indicate that efforts should be made to create new PE-like materials with oxygen functionality to add enzymatic recyclability. Even though PE in nature remains challenging, our study can help guide the field of microbial plastic degradation by highlighting the challenges associated with data interpretation and to advise caution in future studies.

## Supplementary Material

Sandholm_et_al_SupplementaryMaterial_final

Sandholm_et_al_Table_S1_V02_wraf276

Sandholm_et_al_Table_S2_V02_wraf276

Supplementary_Table_Legends_wraf276

## Data Availability

DNA sequencing data (amplicon and shotgun metagenomics) and RNA sequencing data have been deposited at the European Nucleotide Archive (ENA) under the project ID PRJEB97175. The mass spectrometry proteomics data have been deposited to the ProteomeXchange Consortium via the PRIDE (Proteomics Identification Database) partner repository with the data set identifier PXD068679. Source data for SEC, FTIR, Py-GC-MS, NMR, and GC–-MS can be found in Supplementary data. Scripts used for metagenomic, metatranscriptomic, and proteomic analyses can be found at https://github.com/ronjasan/flisa_multiomics. Assembled metagenomes and gene annotations are deposited in Figshare (https://doi.org/10.6084/m9.figshare.30102658 and https://doi.org/10.6084/m9.figshare.30102667, respectively). All data are publicly available as of the date of publication and comply with the data reuse guidelines presented in Hug *et al.* [74].
