# Supplementary material for: Microbial degradation of a widely used model polyethylene is restricted to medium- and long-chain alkanes and their oxidized derivatives
Source: ISME J. 2025 Dec 16;19(1):wraf276. doi: 10.1093/ismejo/wraf276 (PMC12753306; doi:10.1093/ismejo/wraf276)
Supplement: Sandholm_et_al_SupplementaryMaterial_final [file sandholm_et_al_supplementarymaterial_final.pdf]

Supplementary Material for

Microbial degradation of a widely used model polyethylene is restricted to medium- and long-chain alkanes and their oxidized derivatives

Ronja Marlonsdotter Sandholm[1], Gordon Jacob Boehlich[1], Ørjan Dahl[1], Ravindra R. Chowreddy[2], Anton Stepnov[1], Gustav Vaaje-Kolstad[1*] and Sabina Leanti La Rosa[1*]

[1]Faculty of Chemistry, Biotechnology and Food Science, Norwegian University of Life Sciences, 1432 Ås, Norway

[2]Norner Research AS, 3920 Porsgrunn, Norway

*Corresponding authors: sabina.leantilarosa@nmbu.no and gustav.vaaje-kolstad@nmbu.no

Supplementary Materials and Methods

16S rRNA gene Amplicon sequencing of soil samples and data analysis

Soil from the original S1 and S2 samples were sent to DNASense ApS (Aalbor, Denmark) for 16S rRNA gene amplicon sequencing. DNA was extracted using the DNeasy PowerSoil Pro kit (QIAGEN, ID: 47014) following manufacturers' instructions. Size and purity of DNA was assessed using TapeStation 2200 (Agilent, USA) with Genomic DNA and D1000 screentapes. DNA concentration was measured using NanoDrop One Microvolume UV-Vis spectrophotometer (ThermoFisher Scientific) and Qubit dsDNA High Sensitivity Assay (Invitrogen). Amplicon libraries for all kingdoms, covering the V4 and V8 regions of the 16S rRNA gene, were prepared using a custom protocol. To amplify the 16S rRNA gene, 20 ng of DNA was used as a template in the PCR mix with 50 nM of each V4-8 [1,2] specific ([515FB] 5'-GTGYCAGCMGCCGCGGTAA-3'; [1391R] 5'-GACGGGCGGTGWGTRCA-3') primers) following a custom 24 nt barcode sequence for each primer, 0.5 mM dNTP mix and 0.01 U Platinum SuperFi DNA Polymerase (Thermo Fisher Scientific, USA) in the SuperFi Buffer. The gene was amplified using the following program: initial denaturation at 98°C for 3 min, 25 cycles of amplification (98°C for 30 s, 62°C for 20 s, 72°C for 2 min), and a final elongation at 72°C for 5 min. The 16S rRNA gene amplicon libraries were purified using the CleanNGS SPRI beads (CleanNA, NL) with a bead to library ratio of 3:5. Libraries were eluted in 25 µL of nuclease-free H_2O (Qiagen, Germany). Sequencing libraries were then prepared using the Ligation sequencing kit (SQK-LSK114, Oxford Nanopore Technologies, UK), according to manufacturers' protocol with the following modifications: 500 ng total DNA was used as input and CleanNGS SPRI beads were used for library clean-up steps. DNA concentration was measured using Qubit dsDNA HS Assay kit (Thermo Fisher Scientific, USA). Gel electrophoresis using Tapestation 2200 and D1000/High sensitivity D1000 screentapes (Agilent, USA) was used to validate product size and purity. Libraries were sequenced on R10.4 flow cells on a MinION device (Oxford Nanopore Technologies, UK). Reads were basecalled and demultiplexed with MinKNOW guppy g6.4.2 using the super accurate basecalling algorithm (config r10.4.1_400bps_sup.cfg) and the custom 24 nt barcodes.

The SILVA 16S/18S rRNA 138 SSURef NR99 full length database in RESCRIPt format was downloaded from the QIIME on 29th of September 2022 [3-5]. Potential generic place holders and dead-end taxonomic entries were cleared from the taxonomy flat file, i.e. entries containing *uncultured*, *metagenome* or *unassigned*, and were replaced with a blank entry. Sequencing reads in the demultiplexed and basecalled fastq files were filtered for length (320-2000 bp) and quality (phred score > 15) using a local implementation of filtlong v0.2.1 [6] with the settings "–min_length 320 –max_length 2000 –min_mean_q 97". The filtered reads were mapped to the SILVA 138.1 99 % NR database with minimap2 v2.24r1122 [7] using the "-ax

mapont" command and downstream processing using samtools v1.14 [8]. Mapping results were filtered such that query sequence length relative to alignment length deviated < 10%. Noteworthy, low abundant OTUs making up < 0.01% of the total mapped reads within each sample were disregarded as a data denoising step. Further bioinformatic processing was done using R v4.3.3 [9], the R packages tidyverse v2.0.0 [10], microseq v2.1.6 [11], , vegan v2.6-10 [12], BLASTn v2.16.0 [13], and samtools v1.21 [8].

RNA extraction and sequencing of tertiary communities

After RNA extraction, RNA concentration and purity were determined using a Qubit 3.0 fluorometer following the manufacturer's protocol. RNA integrity was checked by using an Agilent 2100 Bioanalyzer (Agilent Technologies, Santa Clara, CA, USA). Samples with a RIN (RNA integrity number) equal to or above 7.0 were processed for library preparation. The library preparation was carried out by Novogene (Beijing, China) using a TruSeq Stranded mRNA kit (Illumina, San Diego, CA, USA), as per the manufacturer's protocol. Strand-specific libraries were sequenced on the Illumina NovaSeq 6000 System (Illumina), and 12 Gb of pair-end 150 bp data per sample was obtained.

Isolation of *Acinetobacter guillouiae* FS11

The S1LMWPE tertiary community was further enriched with MM containing 10 mg/mL low molecular weight PE (LMWPE) before plating onto Lysogeny broth (LB,Sigma-Aldrich, cat. number: L3522) plates supplemented with 50 µg/mL kanamycin sulfate (Gibco, cat. number: 11815-032) and incubation for 48 hours at 30°C. After that, individual colonies were transferred back to MM containing 10 mg/mL LMWPE and grown overnight. Positive cultures were then plated onto LB agar plates with 50 µg/mL kanamycin sulfate and incubated for 2 days at 30°C.

Single colonies were dissolved in 20 µL dH_2O, and 2 µL was used as template DNA for PCR amplification. The reaction included 2.5 µL 10 µM forward primer ([27F] 5´-AGAGTTTGATCMTGGCTCAG-3'), 2.5 µL 10 µM reverse primer ([1492R] 5´-GGTTACCTTGTTACGACTT-3'), 25 µL Q5 High-Fidelity 2X Master Mix (New England Biolabs, cat. number: M0492S) and 18 µL dH_2O. The 16S rRNA gene was amplified using the following program: initial denaturation at 98°C for 2 min, 35 cycles of amplification (98°C for 10 s, 55°C for 30 s, 72°C for 50 s) and a final elongation at 72°C for 10 min. The PCR products were cleaned using the Nucleospin Gel and PCR Clean-up kit (Machery-Nagel, Germany) following manufacturers' protocol. DNA concentration and purity was evaluated using a NanoDrop One Microvolume UV-Vis spectrophotometer (ThermoFisher Scientific, CAT: ND-ONE-W). The amplicons were sent to Eurofins Genomics (Moss, Norway) and sequenced using Sanger sequencing. The resulting sequences were trimmed and assembled into a contig using the R

package sangeranalyseR v1.12.0 [14], and aligned using BLASTn v2.16.0 [13] to the 16S rRNA gene sequences extracted from the MAGs of the S1LMWPE tertiary community.

Proteomics

A. guillouiae FS11 samples for proteomic analysis were processed as follows. LMWPE was separated from the liquid culture by filtering with Whatman 20 µm cellulose filters (Cytiva, USA, WHA10331554). Biofilm was collected from LMWPE by ultrasonication in 20 mM Tris-HCl pH 7.5, 100 mM NaCl, 1% SDS, and 1 mM EDTA. The sonicated cells were centrifuged to remove cell debris, and the supernatant was collected. Planktonic cells were collected from the liquid culture by centrifugation (4,500 x g, 20 min, 4°C), and the supernatant containing the secreted proteins was separated from the pellets. Pelleted cells were lysed using a bead-beating approach, where glass beads (diameter ≤ 106 µm) were added to cells resuspended in lysis buffer (50 mM Tris-HCl pH 7.5, 100 mM NaCl, 0.1% Triton x-100 and 1 mM DTT). The cells were disrupted by three 60s cycles using a FastPrep-24 homogenizer (MP Biomedicals, Santa Ana, CA, USA). The lysate was centrifuged at 16,000 g for 10 min at 4°C to remove cell debris, and the supernatant was collected. Secreted proteins and biofilm-derived proteins were concentrated using Vivaspin 20 (3-kDa molecular weight cutoff) centrifugal concentrators (Sartorius Stedim Biotech GmbH, Germany). Proteins were precipitated in 12.5% trichloroacetic acid (TCA) overnight at 4°C. After overnight precipitation, samples were centrifuged at 15,000 g for 15 min at 4°C, and the pellet was washed in ice-cold wash buffer (10 mM HCl and 90% acetone). After removal of the supernatant, the pellets were air-dried and resuspended in 1X lysis buffer (5% SDS and 50 mM TEAB pH 8.5). S-Trap Mini Columns (Protifi, Fairport, NY, USA) for protein digestion, according to manufacturer's instructions with minor modification. Proteins were reduced using 20 mM dithiothreitol and alkylated with 40 mM iodoacetamine. Proteins were digested using a 1:25 (wt:wt) trypsin (Promeg, cat. number: V5111) to protein ratio. Resulting peptides were analyzed on a nanoLC-MS/MS system, consisting of a nano UHPLC (nanoElute 2, Bruker Daltonics Inc., Bremen, Germany) coupled to a trapped ion mobility spectrometry/quadrupole time of flight mass spectrometer (timsTOF Pro, Bruker Daltonics Inc., Bremen, Germany). Peptides were separated on an Aurora C18 reverse-phase (1.6 µm, 120 Å) 25 cm x 75 µm analytical column with an integrated emitter (IonOpticks, Melbourne, Australia). To keep the temperature of the column at 50°C, the integrated oven was used. Prior to loading samples, the column was equilibrated, with an equilibration pressure of 800 bar. Samples were separated using a solvent gradient from 5% to 25% (solvent B; 0.1% (v/v) formic acid in LC-MS grade acetonitrile) over 70 min, and to 37% over 5 min, with a flow rate of 300 nL/min. Solvent composition was then increased to 95% (solvent B) over 5 min and maintained for an additional 10 min. Solvent A is 0.1% (v/v) formic acid in milliQ water.

The timsTOF Pro was run using positive ion data dependent acquisition PASEF mode, operated with the control software Compass HyStar v6.2.1.13 and timsControl v5.0.4 (30538b33). The mass range for acquisition was set to 100-1700 m/z. The TIMS settings were set to: 1/K0 Start 0.85 V·s/cm2 and 1/K0 End 1.4 V·s/cm2, ramp time 100 ms, ramp rate 9.42 Hz, and duty cycle 100%. Capillary voltage was set at 1400 V, dry gas at 3.0 L/min, and dry temperature at 180°C. MS/MS settings were set to: 10 PASEF ramps, cycle time of 0.53 s, charge range of 0-5, scheduling target intensity at 20 000, intensity threshold at 2500, active exclusion release after 0.4 min, and CID collision energy ranging from 27-45 eV. Raw data were then processed using DataAnalysis v6.1 (build 213.27.0) and the Bruker processing method “Shotgun PASEF ProteinAnalysis2.7comp.m”.

Culturing of *A. guillouiae* FS11 to assess substrate modifications

To obtain samples for GC-MS and SEC analysis, *A. guillouiae* FS11 was cultured in triplicate in MM supplemented with 30 mg/mL LMWPE at 30°C for 7 days. As abiotic control, flasks with MM supplemented with 30 mg/mL LMWPE were set up and subjected to the same condition. At the end of the experiment, the LMWPE powder was separated from the liquid cultures by filtration with Whatman 20 µm cellulose filters (Cytiva, USA, Cat. No: WHA10331554). LMWPE was washed in three consecutive steps to remove biofilm. In the first step, 500 µL dH_2O was added, and the LMWPE was incubated in a ThermoMixer (Eppendorf, Germany) at 1500 rpm for 30 min at RT. This was followed by 10 min of ultrasonication in a Bransonic 3510-DHT Ultrasonic Bath (Emerson, USA), before removing the dH_2O. The cleaning was then repeated using 500 µL 1 M NaOH, then 500 µL 70% ethanol. Samples were air-dried overnight. The control samples were subjected to identical treatment.

Supplementary Results

Microbial communities from a plastic-enriched site include species associated with plastic degradation

Samples S1 and S2 collected from two different locations and depths were subjected to sequencing of the 16S rRNA gene region V4-V8 to determine microbial community composition and diversity. After quality filtering and trimming of sequences, OTU inference, taxonomic identification, removal of contaminant sequences and post clustering curation, we were left with a dataset comprised of 1690 OTUs in S1 and 1946 OTUs in S2, respectively. A rarefaction curve was generated for evaluation of the obtained sequencing depth relative to sample complexity, represented as unique OTUs (**Fig. S1A**). The samples reached saturation, indicating sufficient sampling depth. We analyzed the overall microbiota composition at family level, as 30% of the total ASVs remained unassigned at the genus level. (**Fig. S1B**). The most abundant bacterial families in the S1 microbial community were Xanthobacteraceae (18%),

some unclassified OTUs (13.8%), Thermoanaerobaculaceae (6.9%), Solirubrobacteraceae (3.2%), and Acidobacteria subgroup 2 (3.1%). The S2 community was dominated by some unclassified OTUs (12.7%), the fungal family Amylocorticiaceae (7.5%), Xanthobacteraceae (6.4%), Nitrosomonadaceae (4.7%), and Solirubrobacteraceae (3.8%). The Xanthobacteraceae family is ubiquitous in soil, and has a diverse set of phenotypes, with many members being able to fix N_2, and some strains possess the ability to degrade different alkanes, alkenes, and aromatic compounds [15]. Thermoanaerobaculaceae are also commonly found in soil, and play an important role in nitrogen transformation [16].

In both soils, the families Nocardiaceae, Moraxellaceae displayed moderate abundance. In the S1 soil, the fungal family Hypocreaceae, Herpotrichiellaceae were present, whereas the bacterial family Pseudomonadaceae was found in the S2 soil. Previous research has shown that members of these families have plastic degradation abilities or are colonizers of various types of plastics [17-19]. We then looked at the diversity among the samples from the two locations. Using alpha diversity metrics, both the S1 and S2 soil displayed high diversity (Shannon index: S1, 6.77; S2, 6.87) and differed in microbial composition (Inverse Simpson index: S1: 251; S2, 145). Differences were found on the OTU level, with 333 and 1970 fungal OTUs, and 22 110 and 17 884 bacterial OTUs in the S1 and S2 communities, respectively.

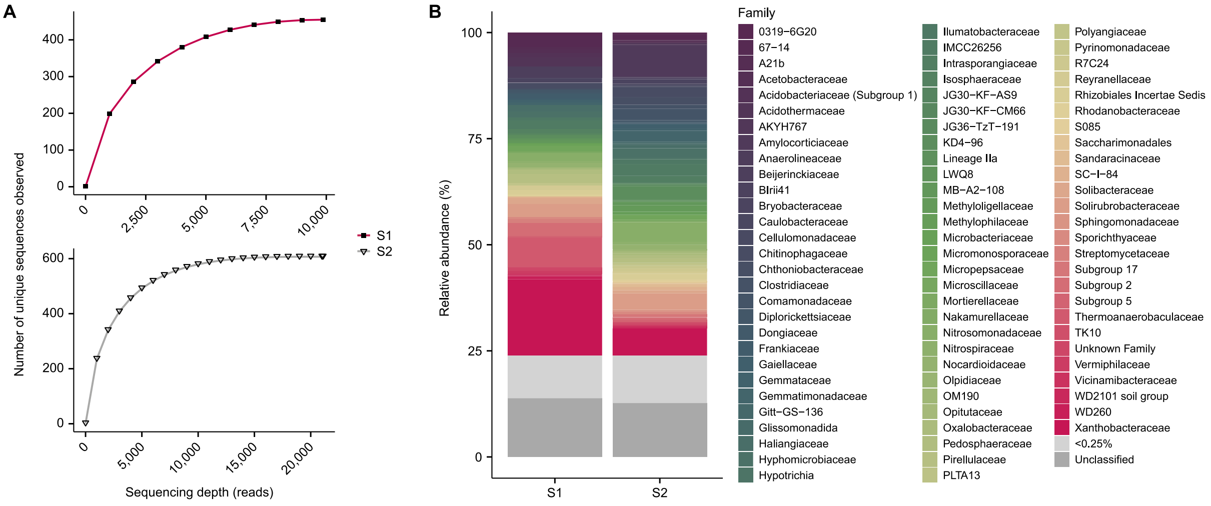

Fig. S1. Species richness and relative abundance of OTUs in S1 and S2. **(A)** Rarefaction curves of the sequenced soil samples S1 and S2. Curves depict sequencing depth relative to sample complexity, quantified as unique OTUs. **(B)** Family-level relative abundance of the taxa detected in S1 and S2. Operational Taxonomic Unit (OTUs) were taxonomically identified by mapping to the SILVA 16S/18S rRNA database. Families with a relative abundance below 0.25% were grouped together, and those not classified by SILVA are labelled as unclassified.

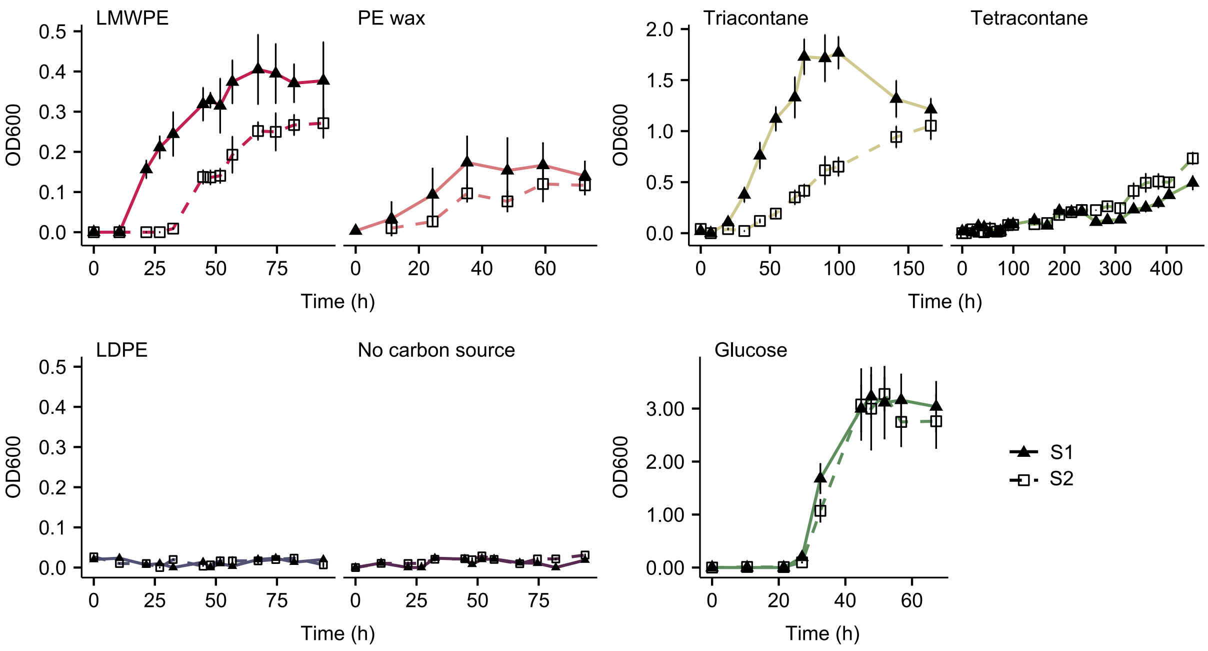

Fig. S2. Growth profiles for the S1 and S2-derived tertiary microbial communities grown on different carbon sources. Communities were grown in minimal salts medium supplemented with 10 mg/mL LMWPE, PE wax, triacontane, tetracontane or low-density PE (LDPE), or 2% (v/v) glucose as the sole carbon source. Data are averages ± standard deviations (error bars) of three biological replicates. Note that the scales for OD600 and time (h) differ between the carbon sources.

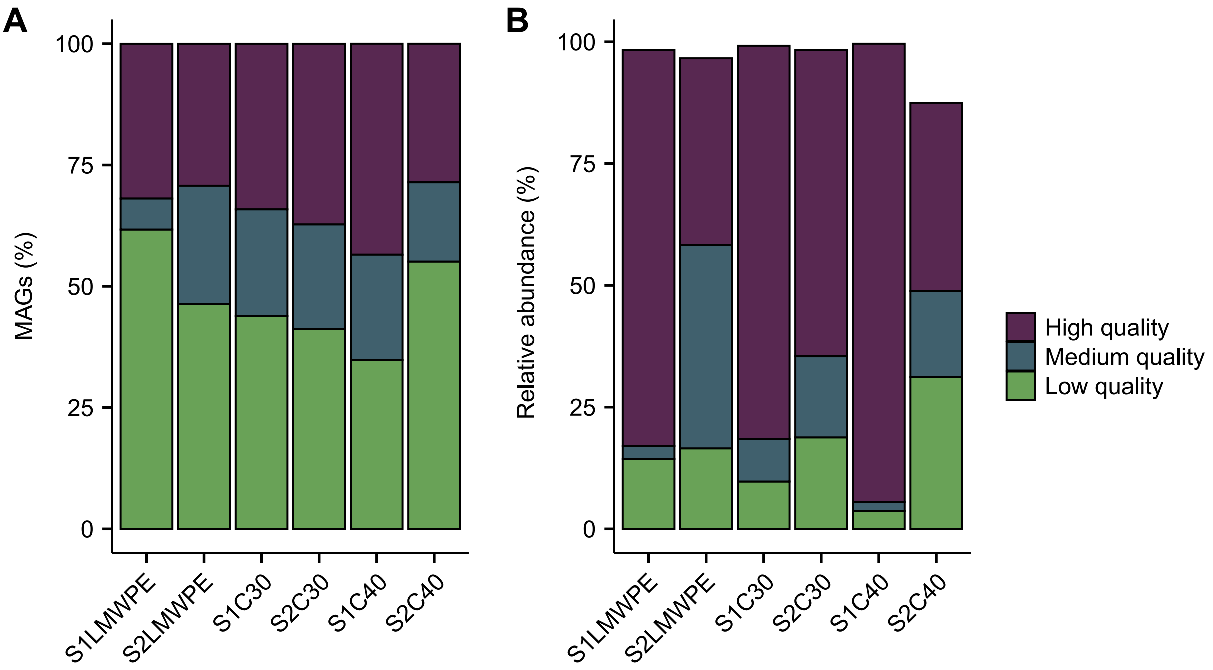

Fig. S3. Quality of the MAGs for each microbial community. **(A)** Relative proportion of quality for the total number of MAGs in each community. **(B)** Relative abundance of MAGs, according to quality. MAG quality was evaluated according to the standard defined by MIMAG [20].

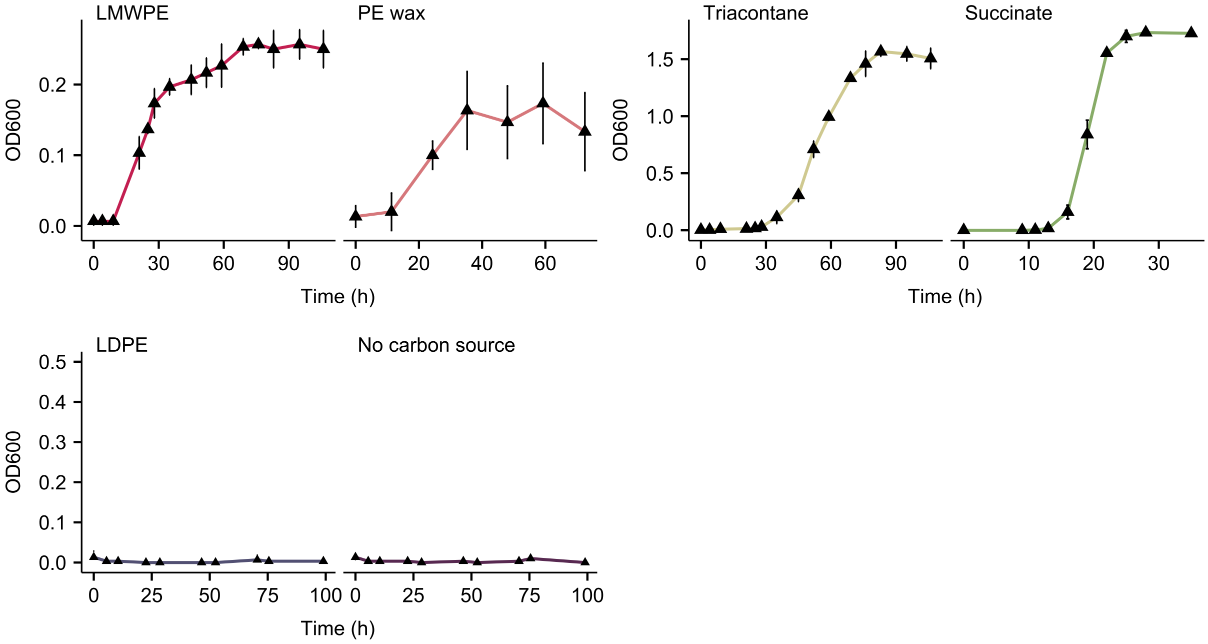

Fig. S4. Growth profiles for *A. guillouiae* FS11 on different carbon sources. The bacterium was grown in minimal salts medium supplemented with 10 mg/mL LMWPE, PE wax, triacontane or low-density PE (LDPE), or 2% (v/v) sodium succinate as the sole carbon source. Data are averages ± standard deviations (error bars) of three biological replicates. Note that the scales for OD600 and time (h) differ between the carbon sources.

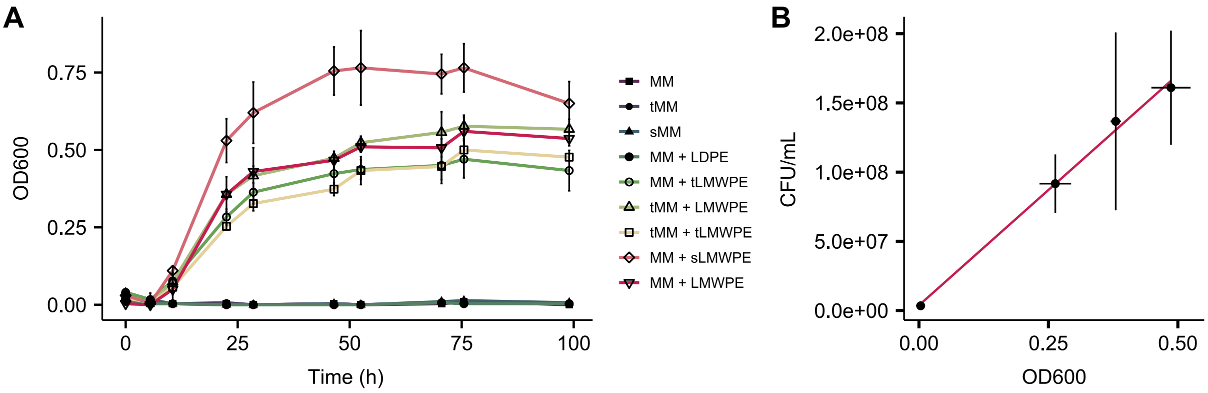

Fig. S5. Cultivation experiments of *A.guillouiae* FS11 on LMWPE and low-density PE (LDPE). **(A)** Growth profiles for *A.guillouiae* FS11 in different control conditions. LMWPE (30 mg/mL) was incubated for a week in MM at 200 rpm at 30°C before being collected for further analysis. The treated incubated substrate and MM (tLMWPE and tMM, respectively) was then combined with fresh medium (MM + tLMWPE) and untreated LMWPE (tMM + LMWPE), as well as together (tMM+ tLMWPE). Untreated LMWPE in fresh MM was subjected to ultrasonication, then separated and combined with fresh MM (MM + sLMWPE. Negative controls, containing no carbon sources, were also prepared. These consist of fresh medium (MM), treated medium (tMM) and medium sonicated with LMWPE (sMM). An abiotic control with fresh MM and LDPE (MM + LDPE) was also included. **(B)** Growth kinetics of *A.guillouiae* FS11 grown on minimal medium (MM) supplemented with 30 mg/mL LMWPE. Colony forming units (CFU) were enumerated by plating aliquots from these cultures onto LB plates.

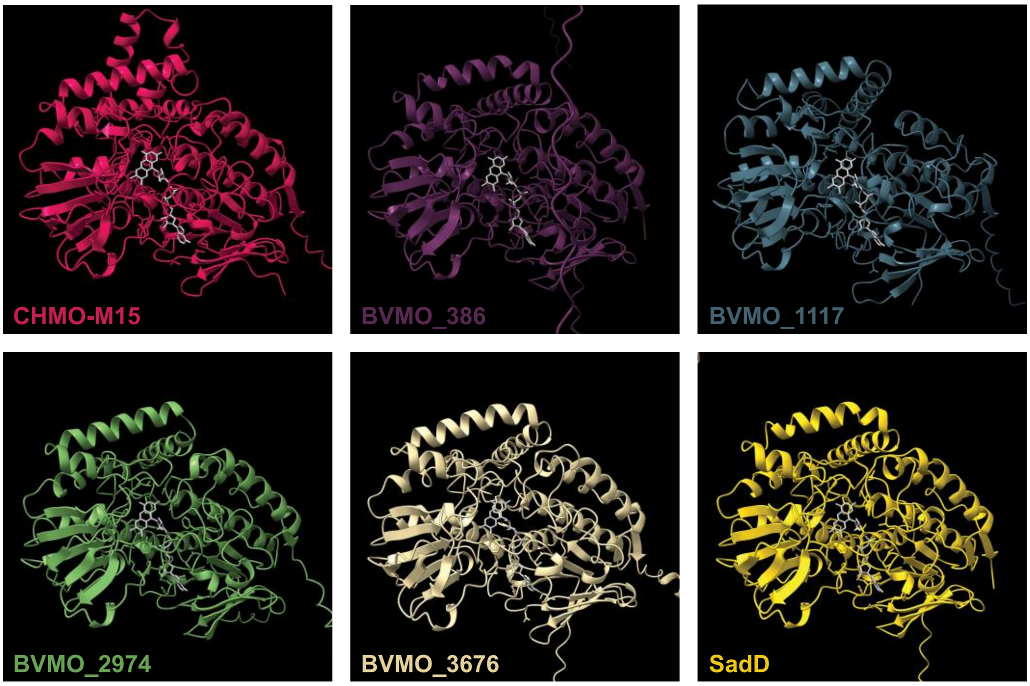

Fig. S6. Predicted structures of *Ag*BVMOs. Structure of two characterized BVMOs, CHMO-M15 [21], and SadD [22], is also shown. FAD structure is depicted in grey.

References

1. Parada AE, Needham DM, Fuhrman JA. Every base matters: assessing small subunit rRNA primers for marine microbiomes with mock communities, time series and global field samples. *Environ Microbiol*. 2016;**18**(5):1403-1414. doi:10.1111/1462-2920.13023

2. Apprill A, McNally S, Parsons R, et al. Minor revision to V4 region SSU rRNA 806R gene primer greatly increases detection of SAR11 bacterioplankton. *Aquat Microb Ecol*. 2015;**75**(2):129-137. doi:10.3354/ame01753

3. Yilmaz P, Parfrey LW, Yarza P, et al. The SILVA and "All-species Living Tree Project (LTP)" taxonomic frameworks. *Nucleic Acids Res*. 2014;**42**(D1):D643–D648. doi:10.1093/nar/gkt1209

4. Robeson MS, O'Rourke DR, Kaehler BD, et al. RESCRIPt: reproducible sequence taxonomy reference database management. *Plos Comput Biol*. 2021;**17**(11):e1009581. doi:10.1371/journal.pcbi.1009581

5. Quast C, Pruesse E, Yilmaz P, et al. The SILVA ribosomal RNA gene database project: improved data processing and web-based tools. *Nucleic Acids Res*. 2012;**41**(D1):D590-D596. doi:10.1093/nar/gks1219

6. Wick R. Filtlong. 2017. Available at https://github.com/rrwick/Filtlong

7. Li H. Minimap2: pairwise alignment for nucleotide sequences. *Bioinformatics*. 2018;**34**(18):3094-3100. doi:10.1093/bioinformatics/bty191

8. Li H, Handsaker B, Wysoker A, et al. The sequence alignment/map format and SAMtools. *Bioinformatics*. 2009;**25**(16):2078-2079. doi:10.1093/bioinformatics/btp352

9. The R Core Team. R: a language and environment for statistical computing. 2024. Available at https://www.R-project.org/

10. Wickham H, Averick M, Bryan J, et al. Welcome to the tidyverse. *J Open Source Softw*. 2019;**4**(43):1686-1692. doi:10.21105/joss.01686

11. Cruickshank R. The microseq package. 2023. Available at https://github.com/larssnip/microseq

12. Oksanen J, Blanchet FG, Friendly M, et al. vegan: community ecology package. 2025. Available at https://CRAN.R-project.org/package=vegan

13. Altschul SF, Gish W, Miller W, et al. Basic local alignment search tool. *J Mol Biol*. 1990;**215**(3):403-410. doi:10.1016/s0022-2836(05)80360-2

14. Chao K-H, Barton K, Palmer S, et al. sangeranalyseR: simple and interactive processing of sanger sequencing data in R. *Genome Biol Evol*. 2021;**13**(3):evab028. doi:10.1093/gbe/evab028

15. Oren A. The family Xanthobacteraceae. In: Rosenberg E, DeLong EF, Lory S, Stackebrant E, Thompson F, eds. *The Prokaryotes: Alphaproteobacteria and Betaproteobacteria*. 4th ed. Springer Berlin Heidelberg; 2014:709-726.

16. Wang M, Xiong W, Zou Y, et al. Evaluating the net effect of sulfadimidine on nitrogen removal in an aquatic microcosm environment. *Environ Pollut*. 2019;**248**:1010-1019. doi:10.1016/j.envpol.2019.02.048

17. Ameen F, Moslem M, Hadi S, et al. Biodegradation of low density polyethylene (LDPE) by mangrove fungi from the red sea coast. *Prog Rubber Plast Re*. 2015;**31**(2):125-143. doi:10.1177/147776061503100204

18. Liu X, Dong X, Wang D, et al. Biodeterioration of polyethylene by *Bacillus cereus* and *Rhodococcus equi* isolated from soil. *Int Microbiol*. 2024;**27**(6):1795-1806. doi:10.1007/s10123-024-00509-7

19. Lyu L, Fang K, Huang X, et al. Polyethylene is degraded by the deep-sea *Acinetobacter venetianus* bacterium. *Environ Chem Lett*. 2024;**22**(4):1591-1597. doi:10.1007/s10311-024-01708-4

20. Bowers RM, Kyrpides NC, Stepanauskas R, et al. Minimum information about a single amplified genome (MISAG) and a metagenome-assembled genome (MIMAG) of bacteria and archaea. *Nat Biotechnol*. 2017;**35**(8):725-731. doi:10.1038/nbt.3893

21. Oiffer T, Leipold F, Süss P, et al. Chemo-enzymatic depolymerization of functionalized low-molecular-weight polyethylene. *Angew Chem*2024;**63**(50):e202415012. doi:10.1002/anie.202415012

22. Yin CF, Xu Y, Li T, et al. Wide distribution of the sad gene cluster for sub‐terminal oxidation in alkane utilizers. Environ Microbiol. 2022;**24**(12):6307-6319. doi:10.1111/1462-2920.16124
