# Supplementary material for: Microbial degradation of a widely used model polyethylene is restricted to medium- and long-chain alkanes and their oxidized derivatives
Source: ISME J. 2025 Dec 16;19(1):wraf276. doi: 10.1093/ismejo/wraf276 (PMC12753306; doi:10.1093/ismejo/wraf276)
Supplement: Supplementary_Table_Legends_wraf276 [file supplementary_table_legends_wraf276.docx]

**Supplementary Table Legends**

**Table S1**. Overview of all MAGs from all communities (taxonomy, quality and relative abundance), as well as differential expression of alkane degradation, 2-ketone oxidation and β-oxidation enzymes in the microbial communities and differential abundance of proteins in the *A. guillouiae* FS11 proteome.

**Table S2**. Source data for SEC, FTIR, Py-GC-MS, NMR and DSC, as well as SEC and GC-MS post-degradation with *A. guillouiae* FS11.
